# The Progress of the Research Defence Society

**Published:** 1909-02-27

**Authors:** 


### The Progress of the Research Defence Society.

A month ago to-day the Research Defence Society celebrated its first anniversary, and in honour of the occasion the Hon. Secretary has issued a circular note reporting the main features of the work and progress of the Society during the first year of its existence. Appropriately enough, the inaugural meeting of the new Dublin branch of this association was held during the last week of January; and the large number of distinguished persons who attended at the Theatre of the Royal Dublin Society, as well as the interest and enthusiasm which animated the proceedings, were worthy alike of the cause and of the Irish capital, and marked with red letters the first birthday of the Research Defence Society. Mr. Stephen Paget, who attended this successful meeting and briefly outlined the methods and efforts of the central committee, points out in his recent report that the Society, by the inauguration of this branch, attained a total membership of more than 2,000. We understand that this figure may already be corrected to 2,240, and that each week sees a further substantial increase, while the proportion of lady members is now larger than ever before. It is a satisfactory and significant fact that, with the steady growth of membership, the proportion of ladies has continued at one in ten; and recent additions indicate that the 10 per cent. of 1908 will be changed to 15 per cent. at the close of the present year. Mr. Paget records various losses which the Society has sustained by death, and tabulates the names of the 31 eminent men and women who have become additional Vice-presidents since he last publicly reported progress nine months ago.

This long and impressive list of Vice-presidents, whose names, many of them, are household words in Art, Literature, Science, and Public Affairs, must be a bitter pill for a certain little group of zealous but misguided officials in whose hands rests the management of the fifteen or so rival anti-vivisection societies. These persons—for all the virulent distrust and enmity with which they favour each other—are yet united in a common endeavour to obstruct the experimental method of research and to vilify all who are concerned therein, and they must therefore be satisfied with the expressive, if somewhat contemptuous, generic title of "Anti-vivisectionist Agitators." We greatly fear that upon these persons any further appeals to their sense of justice or of proportion will be wasted. For it seems to be their business to suppress those facts and arguments which tell against their cause; or if in any given instance that course is impossible, to deny the fact and shout down the argument. By such means they smother the doubts of many well-intentioned people whose subscriptions and legacies are so urgently needed for worthier objects. The first year's operations of the Research Defence Society show, however, that the thinking sections of society—which include some potential anti-vivisectionists—are willing to listen to a calm and reasoned defence of the principles and practice of animal experimentation, and to extend a fair hearing to those fellow creatures whose life-work consists in the advancement of knowledge by methods of scientific research.

For the instruction of honest inquirers the Society has issued eleven pamphlets. These are all of striking interest and bear directly upon the facts of the subject, while several are expressly concerned with nailing to the counter those ancient and dubious tales of horror, those misleading facts and patchwork statistics, which have been circulating too long in company with the genuine coins of anti-vivisection argument. Further valuable pamphlets are already in the course of preparation. Against the straightforward ethical objections to animal experiments which are advanced—under what we consider a mistaken view of the scheme of organised life and a clouded sense of proportion—by certain persons whose intelligence and mental sincerity are beyond question, the Society does not concentrate its attack: its quarrel and ours is not with honest men, however muddleheaded their views may seem to us, but with the industrious scatterers of false and abusive statements, whose day of reckoning fast approaches.

